# Effects of Anesthetics on the Renal Sympathetic Response to Anaphylactic Hypotension in Rats

**DOI:** 10.1371/journal.pone.0113945

**Published:** 2014-11-25

**Authors:** Lingling Sun, Mamoru Tanida, Mofei Wang, Yuhichi Kuda, Yasutaka Kurata, Toshishige Shibamoto

**Affiliations:** 1 Department of Physiology II, Kanazawa Medical University, Uchinada, Ishikawa, Japan; 2 Department of Hematology, the Fourth Affiliated Hospital of China Medical University, Shenyang, China; 3 Department of Colorectal Surgery, the Fourth Affiliated Hospital of China Medical University, Shenyang, China; Universidade Federal do Rio de Janeiro, Brazil

## Abstract

The autonomic nervous system plays an important role in rat anaphylactic hypotension. It is well known that sympathetic nerve activity and cardiovascular function are affected by anesthetics. However, the effects of different types of anesthesia on the efferent renal sympathetic nerve activity (RSNA) during anaphylactic hypotension remain unknown. Therefore, we determined the renal sympathetic responses to anaphylactic hypotension in anesthetized and conscious rats and the roles of baroreceptors in these responses. Sprague-Dawley rats were randomly allocated to anesthetic groups that were given pentobarbital, urethane, or ketamine-xylazine and to a conscious group. The rats were sensitized using subcutaneously injected ovalbumin. The systemic arterial pressure (SAP), RSNA and heart rate (HR) were measured. The effects of sinoaortic baroreceptor denervation on RSNA during anaphylaxis were determined in pentobarbital-anesthetized and conscious rats. In all of the sensitized rats, the RSNA increased and SAP decreased after antigen injection. At the early phase within 35 min of the antigen injection, the antigen-induced sympathoexcitation in the conscious rats was significantly greater than that in the anesthetized rats. Anaphylactic hypotension was attenuated in the conscious rats compared to the anesthetized rats. The anesthetic-induced suppression of SAP and RSNA was greater in the order ketamine-xylazine >urethane = pentobarbital. Indeed, in the rats treated with ketamine-xylazine, RSNA did not increase until 40 min, and SAP remained at low levels after the antigen injection. The baroreceptor reflex, as evaluated by increases in RSNA and HR in response to the decrease in SAP induced by sodium nitroprusside (SNP), was suppressed in the anesthetized rats compared with the conscious rats. Consistent with this finding, baroreceptor denervation attenuated the excitatory responses of RSNA to anaphylaxis in the conscious rats but not in the pentobarbital-anesthetized rats. RSNA was increased markedly in conscious rats during anaphylactic hypotension. Anesthetics attenuated this antigen-induced renal sympathoexcitation through the suppression of baroreceptor function.

## Introduction

Anaphylactic shock triggered by an allergic reaction is potentially life threatening. Systemic hypotension triggers the baroreceptor reflex system, resulting in an increase in sympathetic nerve outflow to increase the decreased blood pressure. The sympathetic nervous system has been shown to respond to anaphylactic hypotension in dogs [1],[2] and pigs [3]. In anesthetized rats, sympathetic activity to the kidney was stimulated during anaphylaxis [4]. In addition, we recently reported that either inhibition of the α-adrenoceptor, which is the primary receptor for norepinephrine, or chemical sympathectomy with 6-hydroxy-dopamine attenuated the blood pressure recovery during the late phase of rat anaphylactic hypotension [5]. These lines of evidence suggest that the sympathetic nervous system plays an important role in regulating blood pressure during anaphylactic hypotension.

We previously reported that the heart rate (HR) response to anaphylactic hypotension differed between anesthetized rats and conscious rats: tachycardia was induced in conscious rats but not in anesthetized rats [6]. Consistent with this finding, the sympathetic nerve outflows and cardiovascular function in rats were affected by anesthetics: the increases in renal sympathetic nerve activity (RSNA) and blood pressure induced by electrical or chemical stimulation of the region around the central chemoreceptors in conscious rats were attenuated under pentobarbital anesthesia [7],[8]. In addition, previous studies reported that he RSNA in conscious rats was decreased by an acute injection of urethane [9] and that the blood pressure and HR in spontaneously hypertensive rats were reduced under ketamine-xylazine anesthesia [10]. Thus, it seems likely that RSNA during anaphylactic hypotension may be influenced by the type of anesthetic. However, the effects of different types of anesthesia on RSNA during anaphylactic hypotension have not been investigated. In addition, the renal sympathetic response to anaphylaxis in free-moving conscious rats has not been determined.

The previous study reported that an increase in RSNA during anaphylactic hypotension is partly mediated by baroreceptor signaling in pentobarbital-anesthetized rats [4]. However, the roles of the baroreceptor reflex in anaphylactic hypotension in conscious rats remain unknown.

Therefore, the primary purpose of present study was to determine the renal sympathetic response to anaphylactic hypotension in rats anesthetized with different anesthetics, pentobarbital, urethane or ketamine-xylazine, and in conscious rats. Second, we determined whether the renal sympathetic response to anaphylactic hypotension depends more strongly on the inputs from the sinoaortic baroreceptors in conscious rats than in pentobarbital-anesthetized rats.

## Materials and Methods

### Animals

Male Sprague-Dawley rats, weighing 350–380 g, were housed in a room maintained at 24±1°C and illuminated for 12 h (08:00–20:00) every day. Food and water were freely available. All animal care and handling procedures were approved by the Animal Research Committee of Kanazawa Medical University.

### Sensitization

Rats were sensitized by a subcutaneous injection of an emulsion made by mixing equal volumes of complete Freund's adjuvant (0.5 ml) with 1 mg of ovalbumin (grade V, Sigma St. Louis, USA) dissolved in physiologic saline (0.5 ml) [11]. Non-sensitized rats were injected with complete Freund's adjuvant and ovalbumin-free saline.

### Anesthetized rats with an intact neuraxis

We examined the effects of an intraperitoneal administration of three types of anesthetics: urethane (1.2 g/kg), pentobarbital sodium (50 mg/kg), and a ketamine and xylazine cocktail (91 mg/kg and 9.1 mg/kg, respectively). The doses of these anesthetics were determined by referring to previous studies [5],[10],[12]. While the rats were under each type of anesthesia, polyethylene catheters were inserted into the right jugular vein and the left femoral artery for intravenous injections and for the systemic arterial pressure (SAP) recording, respectively. The animal was then cannulated through the trachea and fixed in a stereotaxic apparatus. Body temperature was maintained at 37.0–37.5°C using a heating pad and was monitored using a thermometer inserted into the rectum. RSNA was measured as in our previous study [12]. In brief, the left renal nerve was exposed retroperitoneally through a left flank incision using a dissecting microscope. The nerve was attached to a pair of stainless steel wire electrodes and then connected to electrodes for RSNA recordings. The recording electrodes were fixed with a silicone gel (liquid A & liquid B, Kagawa kikai Co. JAPAN) to prevent dehydration and for electrical insulation.

The electrical change in the renal nerve was amplified 20,000–50,000 times with a bandpass of 100 to 1,000 kHz and was monitored by an oscilloscope. The raw data of the nerve activity were converted to standard pulses by a window discriminator. Both the discharge rate and the neurogram were sampled with a Power-Lab analog-to-digital converter for recording and data analysis on a computer. The background noise, which was determined 30–60 min after the animal was euthanized, was subtracted. The nerve activity was rectified and integrated, and the baseline nerve activity was normalized to 100%. SAP and HR were also sampled using Power-Lab and were stored on a hard disk for off-line analysis.

After surgery, each rat was allowed to stabilize for 10–30 min and was then injected intravenously with a bolus of sodium nitroprusside (SNP: 30 µg/kg) to induce baroreceptor-mediated sympathoexcitation. Next, the baseline was measured for 5 min prior to the intravenous injection of the antigen: ovalbumin (0.6 mg). The parameters were recorded for 60 min after the antigen injection. At the end of the experiment, hexamethonium chloride (10 mg/kg) was intravenously administered to ensure that the post-ganglionic efferent sympathetic nerve activity was recorded.

### Conscious rats with an intact neuraxis

To examine the RSNA response to anaphylactic hypotension in conscious rats, free-moving instrumented rats were used. While the rats were under pentobarbital anesthesia (50 mg/kg, ip), polyethylene catheters were inserted into the right jugular vein and the left femoral artery for intravenous injections and SAP recording, respectively. In a manner similar to that for the anesthetized rats, the left renal nerve was connected to electrodes to record nerve activity. The recording electrodes were fixed with a silicone gel (Kwik-Sil; WPI Inc. Sarasota, USA) to prevent dehydration and for electrical insulation. The lead wires from the recording electrodes and two catheters were tunneled under the skin to exit through the nape of the neck and were fixed to the skull using dental cement. The RSNA was measured during recovery from anesthesia and after the awakening. The rats were allowed to recover for at least 48 h after the implantation surgery to minimize the possible effects of surgical stress. The measurements and the administration of SNP and antigen were performed in the same manner as for the anesthetized rat experiment.

### Anesthetized and conscious rats with sinoaortic denervation

Bilateral sinoaortic denervation (SAD) was performed 3–5 days before the experiments involving the intravenous administration of SNP and antigen. While the rats were under pentobarbital anesthesia (50 mg/kg, ip) and after the neurovascular trunk in the neck was exposed, the carotid sinus, aortic depressor and recurrent laryngeal nerves were sectioned bilaterally to complete the baroreceptor denervation. The rats were allowed to recover for 3–5 days. For anesthetized sham rats, the neck was exposed without sectioning the nerves. Then, the rats with SAD were divided into the pentobarbital-anesthesia group and the conscious group. The conscious sensitized rats with an intact neuraxis, as described above, served as the conscious sham rats. The measurements and the administration of SNP and antigen were performed in the same manner as for the anesthetized or conscious rat experiments. Successful baroreceptor denervation was confirmed by the absence of reflex increases in HR and RSNA in response to the decreased blood pressure induced by an injection of SNP.

### Data Analysis

All data are expressed as means ±SEM. Percent changes from baseline values were calculated for RSNA. When comparing the baseline levels between groups and the responses of the sympathetic and hemodynamic parameters to the antigen or SNP between groups, ANOVA with the Bonferroni post hoc test was applied. P<0.05 was considered statistically significant.

## Results

### Responses to anaphylaxis in conscious and anesthetized rats

**Figure 1:**
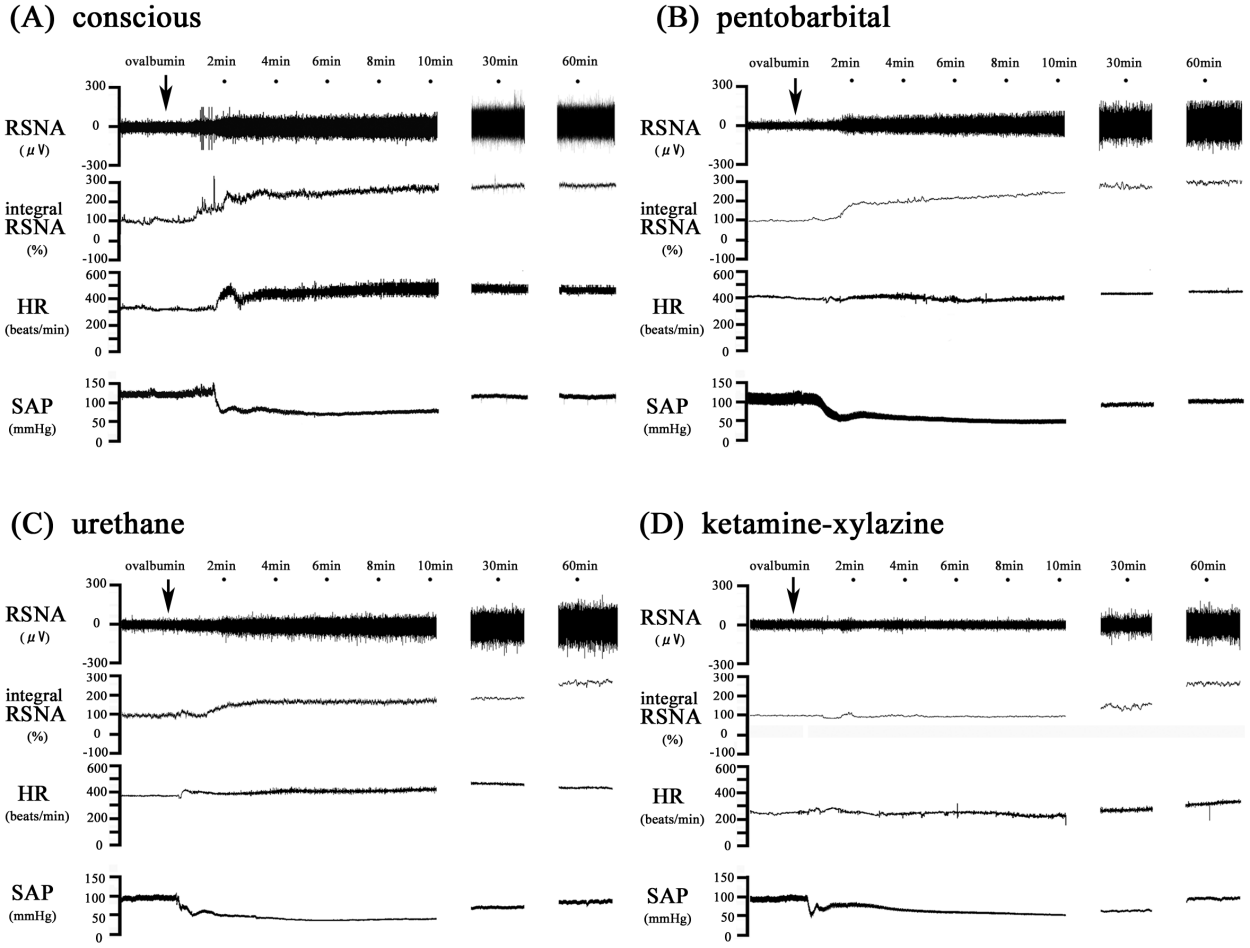
Sympathetic and hemodynamic responses to anaphylaxis in conscious and anesthetized rats. Representative recordings of RSNA, SAP and HR after an intravenous injection of the antigen, ovalbumin, into a sensitized conscious rat (A) and a sensitized rat under anesthesia with pentobarbital (B), urethane (C) or ketamine-xylazine (D).

**Figure 2:**
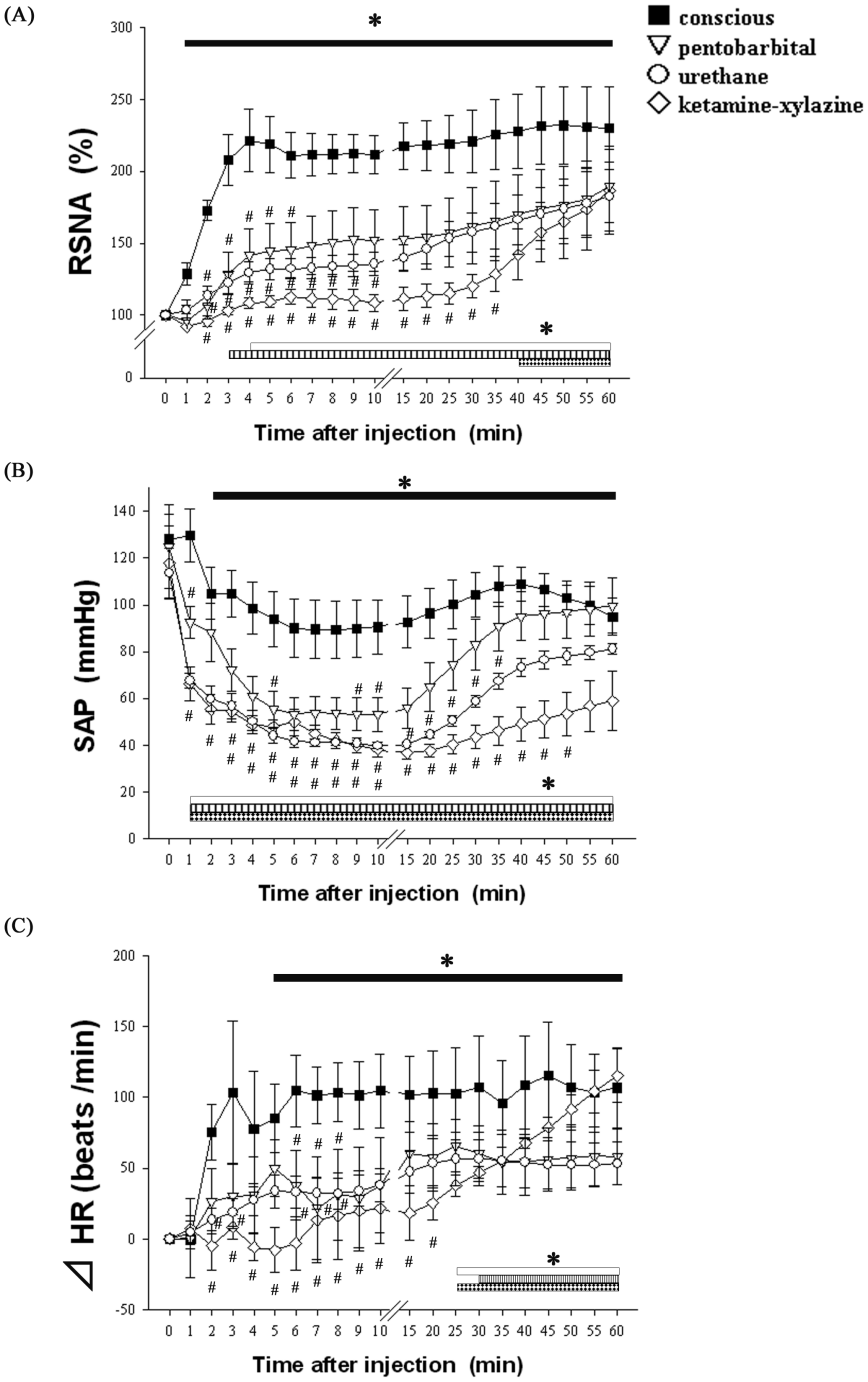
Sympathetic and hemodynamic responses to anaphylaxis in conscious and anesthetized rats. Time-course data of the changes in RSNA (A), SAP (B) and HR (C) after an intravenous injection of the antigen, ovalbumin, into the sensitized rats under anesthesia with pentobarbital (▽, n = 6), urethane (▿, n = 6) or ketamine-xylazine (○, n = 6) and into the sensitized and conscious rats (▪, n = 5). Values are expressed as the mean + SEM. #P<0.05 vs. the conscious rats. *P<0.05 vs. baseline values; The black bar, white bar, vertical-striped bar or black bar with white diamonds show the significant changes from the corresponding baseline values in the conscious, pentobarbital-, urethane- or ketamine-xylazine-anesthetized rats, respectively.

We first examined the RSNA, SAP and HR responses to the antigen injection in anesthetized and conscious rats (Fig. 1). In all sensitized rats with an intact neuraxis, the antigen injection evoked an increase in RSNA and a decrease in SAP (Fig. 2A and 2B). Notably, the RSNA in the conscious rats increased rapidly after the antigen injection, reaching a high level of 221+21% of the baseline at 4 min and then remaining elevated until 60 min. In contrast, in both of the anesthetic groups, the RSNA began to increase more gradually and reached the peak levels at the end of experimental period. Indeed, in the ketamine-xylazine group, the RSNA did not significantly increase until 40 min after the antigen injection, whereas significant increases were observed at 3 and 4 min in the urethane and pentobarbital groups, respectively (Fig. 2A).Significant differences in RSNA were found during the early phase after antigen injection, i.e., 2–5 min for the pentobarbital group, 2–10 min for the urethane group, and 2–35 min for the ketamine-xylazine group, compared with that in the conscious rats (Fig. 2A). This finding suggests that the potency to inhibit RSNA was greater in the order ketamine-xylazine > urethane  =  pentobarbital. Although SAP decreased after the antigen injection in all of the sensitized rats, the hypotensive response in the conscious rats was significantly smaller than that in all of the anesthetized rats (Fig. 2B). Among the anesthetized groups, the severity of the SAP decrease was greater in the order ketamine-xylazine > urethane > pentobarbital, as evidenced by the nadir levels of 36, 39, and 53 mmHg, respectively. Furthermore, the SAP levels 60 min after the antigen injection were smaller in the order ketamine-xylazine (58 mmHg) <urethane (81 mmHg) <pentobarbital (99 mmHg). The HR in the conscious rats increased in a manner similar to the RSNA: the HR increased rapidly, reaching a peak 3 min after the antigen injection and then remaining at this elevated level (Fig. 2C). In contrast, in both types of anesthetized rats, the HR did not increase until 25–30 min after the antigen injection. There were significant differences in HR during the early phase (within 8 min of antigen injection) between the conscious rats and each type of anesthetized rats (Fig. 2C). In contrast, in the non-sensitized rats of either group, the RSNA, SAP and HR did not significantly change after the antigen injection (data not shown).

### Responses to SNP-induced hypotension in anesthetized rats and conscious rats

**Figure 3:**
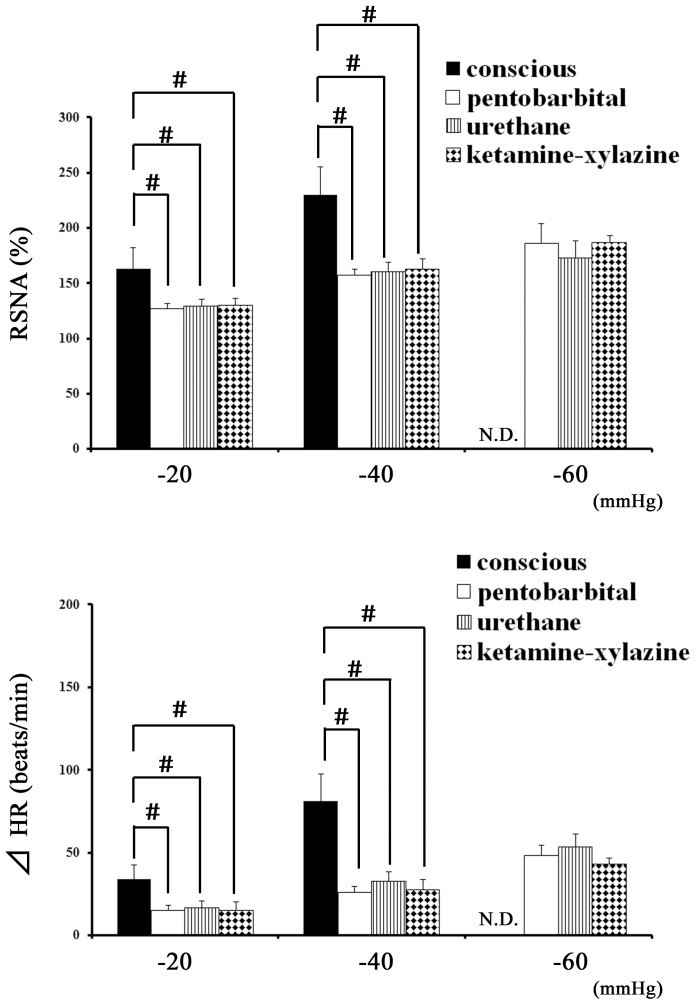
RSNA and HR responses to hypotension with an intravenous injection of SNP in conscious and anesthetized rats. RSNA (A) and HR (B) responses to the decrease in blood pressure (−20, −40 and −60 mmHg) induced by the SNP injections in the conscious rats (black bar, n = 5) and the rats anesthetized with pentobarbital sodium (white bar, n = 6), urethane (vertical-striped bar, n = 6) or ketamine-xylazine (black bar with white diamond, n = 6). Values are expressed as the mean +SEM. N.D.; data were not obtained because SAP did not decrease by 60 mmHg in conscious rats. #P<0.05 vs. the conscious rats.

We further examined the RSNA and HR responses to an SNP injection in anesthetized and conscious rats to evaluate the baroreceptor reflex. In the normal baroreceptor reflex, the decrease in SAP in response to an SNP injection reflexively increases the RSNA and HR via baroreceptor unloading. We analyzed the relation of RSNA and HR to SAP when the SAP was decreasing by 20, 40 and 60 mmHg. In the conscious rats, the SAP did not decrease by 60 mmHg after the SNP injection. In the rats anesthetized with pentobarbital, urethane, or ketamine-xylazine, the excitatory responses of RSNA (Fig. 3A) and HR (Fig. 3B) to the SNP-induced hypotension (−20, −40) were significantly smaller than those in the conscious rats. There were no significant differences in the RSNA or HR among the anesthetic groups (Fig. 3A and 3B).

### Effect of baroreceptor denervation on the responses to anaphylaxis and SNP in conscious and pentobarbital-anesthetized rats

**Figure 4:**
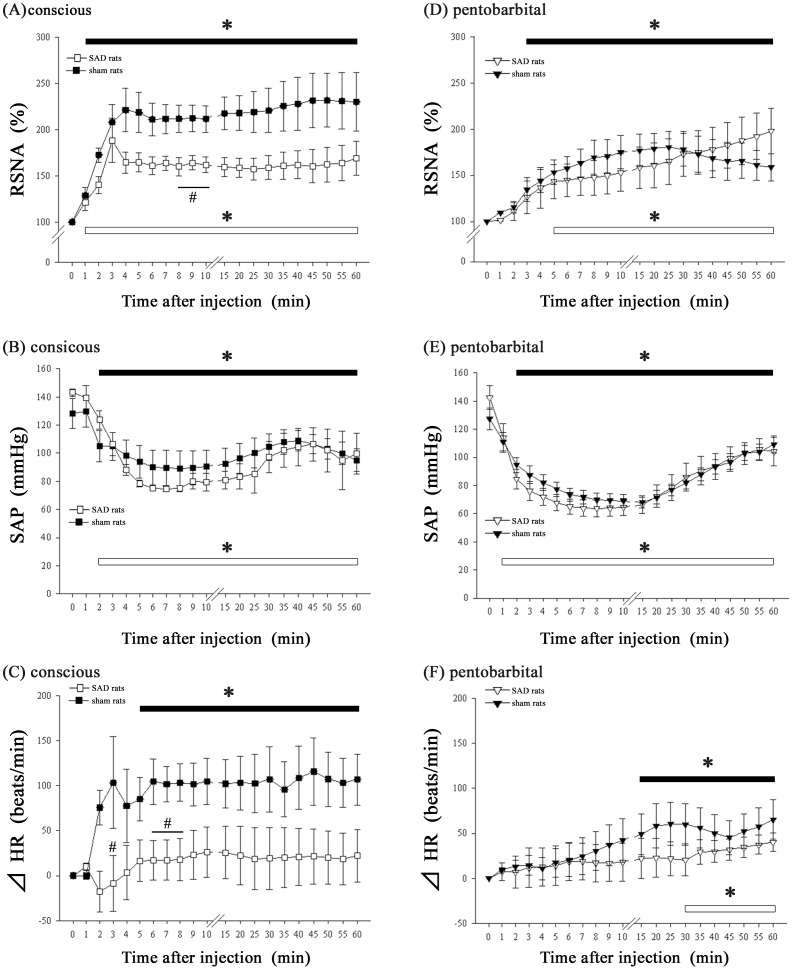
Effects of SAD on the RSNA, SAP, and HR after the antigen injection in the conscious rats and the pentobarbital-anesthetized rats. Time-course data of the changes in RSNA (A), SAP (B) and HR (C) after an intravenous injection of the antigen, ovalbumin, in the sensitized sham rats (▪, n = 5) and the sensitized SAD rats (□, n = 5) that were conscious. Time-course data of the changes in RSNA (D), SAP (E) and HR (F) after an intravenous injection of the antigen, ovalbumin, in the sensitized sham (▾, n = 5) and the sensitized SAD rats (▽, n = 6) under pentobarbital sodium anesthesia. Values are expressed as the mean +SEM. #P<0.05 vs. the sensitized sham rats. *P<0.05 vs. baseline values; The black and white bars show significant changes from corresponding baseline values in the sham and SAD rats, respectively.

In both the conscious sham and SAD rats, the RSNA rapidly increased to approximately 200% of the baseline 3 min after the antigen injection. However, the increase in RSNA during the early phase of anaphylaxis (8–10 min after antigen injection) in the SAD rats was significantly smaller than that in the sham rats (Fig. 4D). Consistently, the SAP in the SAD rats tended to be lower during the early phase than that in the sham rats (Fig. 4E). After antigen injection, the HR significantly increased in the sham rats, but it did not change in the SAD rats (Fig. 4F). Thus, in the conscious rats, SAD attenuated the antigen-induced increases in RSNA and HR.

In contrast to conscious rats, both the sham and SAD rats that were under pentobarbital anesthesia showed similar increases and decreases in RSNA and SAP, respectively, after antigen injection, and no significant differences were found between these two groups (Fig. 4A and 4B). Although the HR was increased significantly from the baseline during the late phase after antigen injection in both the sham and SAD rats, there were no significant differences between the sham and SAD rats (Fig. 4C). These findings suggest that the baroreceptor reflex was suppressed in the anesthetized rats.

We confirmed successful SAD in both the conscious and anesthetized SAD rats, as evidenced by the absence of the renal sympathoexcitatory response to SNP-induced hypotension (109±1% at −20 mmHg and 116±2% at −40 mmHg in conscious rats; 100±2% at −20 mmHg and 103±1% at −40 mmHg in anesthetized rats).

## Discussion

In the present study, we obtained three major findings: 1) RSNA and HR substantially increased in conscious rats during anaphylactic hypotension, while these excitatory responses were attenuated by anesthetics in the order ketamine-xylazine > urethane  =  pentobarbital. Anaphylactic hypotension in conscious rats was less severe than that in anesthetized rats. 2) In anesthetized rats, the baroreceptor reflex, as evaluated by the excitatory responses of RSNA and HR to the decreased SAP induced by SNP, was suppressed compared with that in conscious rats. 3) SAD attenuated the antigen-induced increases in RSNA and HR during the early phase of anaphylactic hypotension in conscious rats but not in pentobarbital-anesthetized rats. Taken together, these findings indicate that RSNA and HR substantially increase in conscious rats during anaphylactic hypotension and that anesthetics attenuate these excitations and worsen anaphylactic hypotension. In addition, the baroreceptor reflex control of RSNA and HR was suppressed during the SNA-induced hypotension and anaphylaxis by the anesthetics. Thus, the anesthetic-associated suppression of baroreceptor function may partly explain why the anesthetics attenuated the elevations of RSNA and HR, as observed in the conscious rats during anaphylactic hypotension.

Previously, Potas et al. reported that RSNA was activated during anaphylaxis in anesthetized rats [4]. In contrast, in anesthetized dogs, RSNA did not increase consistently in response to anaphylaxis [1]. However, it remained unknown whether the RSNA response to anaphylaxis was different in conscious animals. To the best of our knowledge, we provide the first report that conscious rats showed a greater increase in RSNA during anaphylactic hypotension than that of any of the rats anesthetized with pentobarbital, urethane, or ketamine-xylazine (Fig. 2).

Using the same anaphylaxis model, we previously reported that the sympathetic nervous system played significant roles in the late recovery phase (40–60 min after antigen), but not in the early phase of decreasing blood pressure (1–10 min after antigen), of anaphylactic hypotension in pentobarbital-anesthetized rats: chemical sympathetectomy and alpha-adrenoceptor blockade suppressed the blood pressure recovery during the late phase [5]. The present result on the RSNA response to anaphylaxis in pentobarbital-anesthetized rats is consistent with this previous finding: RSNA increased only slightly during the early phase, while it progressively and markedly increased during the late phase after 20 min, reaching peak levels of 189+25% of the baseline at 60 min after antigen injection (Fig. 2A).

In the present study, anesthesia attenuated the excitatory response of RSNA and HR to anaphylactic hypotension, as observed in conscious rats. However, the mechanism for this sympathoinhibition by anesthetics is unknown. The first possibility is related to the changes in the basal levels of RSNA by anesthetics. Generally, anesthetics affect the basal levels of sympathetic nerve activity, blood pressure [9],[10],[13] and the activity of neurons in the medulla oblongata [14], which plays a critical role in the control of cardiovascular function. However, this hypothesis is not likely because the basal level of RSNA in the conscious rats was similar to that in any of the anesthetized rats (Table 1). Another more plausible explanation is the anesthetic-induced suppression of the baroreceptor reflex control of RSNA and HR. As shown in Fig. 3A and 3B, the rats under anesthesia with any of the anesthetics showed a smaller baroreceptor reflex than that of the conscious rats: the reflex increases in RSNA and HR induced by the SNP-induced hypotension in any of the anesthetized rats were significantly smaller than those in the conscious rats. This result is consistent with the finding of no significant differences in the RSNA response to anaphylaxis between the rats with an intact neuraxis and those with SAD (Fig. 4A). If the baroreflex had functioned normally in the anesthetized rats, the post-antigen levels of RSNA in the intact anesthetized rats would have been higher and comparable to those of the intact conscious rats, as shown in Fig. 4A. However, the baroreceptor reflex was depressed by pentobarbital [15] and urethane [16].

**Table 1:** The basal levels of the RSNA, SAP and HR before the antigen injection.

	groups	RSNA (µv.s)	SAP (mmHg)	HR (beats/min)
*anesthetized*				
pentobarbital	non-sensitized (n = 6)	84.0±14.7	129.7±8.2	380±27
	sensitized (n = 6)	92.5±11.3	124.5±6.9	401±22
	sham sensitized (n = 5)	73.3±27.4	127.4±7.7	377±17
	SAD sensitized (n = 5)	88.3±12.4	142.6±8.5	387±15
urethane	non-sensitized (n = 6)	93.9±31.2	117.7±2.6	354±39
	sensitized (n = 6)	102.7±23.1	113.7±2.4	416±22
ketamine-xylazine	non-sensitized (n = 6)	107.8±20.1	124.2±7.2	276±13^a^^,^ b ^,^ c
	sensitized (n = 6)	84.4±11.6	118.0±5.9	255±28^a^^,^ b ^,^ c
*consicous*	non-sensitized (n = 5)	82.6±8.5	117.3±2.8	412±20
	sensitized (n = 5)	78.4±9.0	128.1±8.8	354±39
	SAD sensitized (n = 5)	100.6±22.1	143.1±2.8	416±22

RSNA; renal sympathetic nerve activity, SAP; systemic arterial pressure, HR; heart rate, Values are expressed as means +SE.

a ; P<0.05 vs. the pentbabital rats.

b ; P<0.05 vs. the urethane rats.

c ; P<0.05 vs. the consious rats.

The mechanism for the anesthetic-related suppression of the baroreceptor reflex is not clear, but some possibilities can be provided. Anesthetics may change the sensitivity of the neurons in the rostral ventrolateral medulla (RVLM), an important brainstem region involved in the generation of the sympathetic activity to the cardiovascular system and in the central pathway of the baroreceptor reflex. Indeed, pentobarbital sodium may affect the RVLM as well as the nucleus tractus solitarius by potentiating GABAergic neurotransmission, resulting in inhibition of the activity of the RVLM and the nucleus tractus solitarius. GABAergic neurons have been located in the RVLM [17], and pentobarbital functions as a GABA agonist by acting on GABA receptors [18]. Urethane also affects the RVLM, as indicated by reports that urethane evoked a larger number c-Fos-positive neurons in the RVLM [19]. Thus, anesthetics may inhibit the primary area, the RVLM, in the baroreceptor reflex pathway of the central nervous system, resulting in attenuation of the baroreceptor reflex.

In the anesthetized groups of the present study, ketamine-xylazine more strongly suppressed the RSNA excitation induced by anaphylaxis than did pentobarbital and urethane; significant increases in RSNA were found within 4 min after antigen injection in the pentobarbital and urethane groups but not until 25 min in the ketamine-xylazine group (Fig. 2A). Furthermore, in the sensitized rats under ketamine anesthesia, the RSNA at 10 min after antigen injection (109±6%) was comparable to that of the nonsensitized rats (97±4%) without anaphylactic hypotension. The mechanism for the decreased RSNA excitatory response under ketamine-xylazine compared with that under pentobarbital or urethane is not known. However, a difference in baroreflex sensitivity seems unlikely because the excitatory responses of RSNA and HR to the SNP-induced hypotension were similar among all of the anesthetic groups (Fig. 3). Another possibility is related to the properties of the α2-adrenoceptor. Xylazine exerts an α2-adrenoceptor agonist effect [20],[21] in the central nervous system, thus inhibiting sympathetic outflow [20],[21]. Consistent with this assumption, our data showed that the basal HR in the ketamine-xylazine rats was significantly smaller than that in the pentobarbital and urethane rats (Table 1).

In the present study, the RSNA increased similarly with and without SAD denervation during the anaphylactic hypotension in the pentobarbital-anesthetized rats (Fig. 4A). In addition, the conscious rats with SAD also showed RSNA excitation after the antigen injection (Fig. 4D). These findings suggest that there was a baroreflex-independent excitation of RSNA in the conscious rats. There are several explanations for the antigen-induced RSNA excitation, which is independent of the baroreflex. First, central chemoreceptors in the brainstem, which have excitatory effects on sympathetic outflow in response to high levels of CO_2_ or acidemia [22], might be activated, resulting in RSNA excitation during anaphylaxis. Moreover, it was reported that anaphylaxis stimulated the CO_2_ production of mast cells [23]. Furthermore, the acute severe hypotension induced by anaphylaxis might cause tissue anoxia and the resultant acidemia. Second, chemical mediators are produced as a result of the anaphylaxis, such as histamine, prostaglandins and interleukins, and have excitatory effects on sympathetic outflow [24],[25],[26]; these mediators might directly act on the central nervous system to enhance the RSNA in anesthetized rats. In fact, we previously demonstrated that the injection of histamine into the hypothalamus increased RSNA in a dose-dependent manner in anesthetized rats [24]. Moreover, the central injection of interleukins [25] or [26] prostaglandins also accelerated the sympathetic nerve activity in anesthetized rats. Thus, these lines of evidence suggest that central chemoreceptors or chemical mediators might increase RSNA during anaphylactic hypotension without baroreceptor mediation in conscious SAD rats.

In summary, in response to anaphylactic hypotension, RSNA and HR increased considerably in conscious rats. Anesthetics attenuated these anaphylaxis-induced increases in RSNA and HR in the order xylazine-ketamine > urethane  =  pentobarbital. SAD attenuated the antigen-induced increase in RSNA and HR in conscious rats but not in anesthetized rats. This result indicates that the baroreceptor reflex functioned well in the conscious rats but not in the anesthetized rats. The impairment of the baroreceptor reflex under anesthesia was confirmed by the finding of smaller increases in RSNA and HR in response to the hypotension induced by SNP in the anesthetized rats. Finally, the absence of the normal baroreceptor reflex under anesthesia may account, at least in part, for the decreased RSNA excitation during the anaphylactic hypotension in the anesthetized rats. Serious anaphylactic shock occurring under anesthesia is rare, but can rapidly evolve into life-threatening situations if not recognized and managed promptly [27]. The present results indicated that the beneficial response of sympathoexcitation, as observed in conscious rats with anaphylaxis, was suppressed by anesthetics through attenuation of the baroreceptor reflex, aggravating anaphylactic hypotension under anesthesia. This finding suggests that surgeons and anesthesiologists should treat much carefully the patients suffering from anaphylaxis under anesthesia.
