# Correction: Effects of Anesthetics on the Renal Sympathetic Response to Anaphylactic Hypotension in Rats

**DOI:** 10.1371/journal.pone.0118042

**Published:** 2015-02-11

**Authors:** The PLOS ONE Staff

In the Funding section, the information for author TS is incorrect. The corrected statement should read: TS is supported by a Grant from Kanazawa Medical University (SR2012-04).
